# Characterization of Dextran Produced by the Food-Related Strain *Weissella cibaria* C43-11 and of the Relevant *Dextransucrase* Gene

**DOI:** 10.3390/foods11182819

**Published:** 2022-09-13

**Authors:** Palmira De Bellis, Massimo Ferrara, Anna Rita Bavaro, Vito Linsalata, Mariaelena Di Biase, Biagia Musio, Vito Gallo, Giuseppina Mulè, Francesca Valerio

**Affiliations:** 1 Institute of Sciences of Food Production (ISPA), National Research Council (CNR), Via G. Amendola 122/O, 70126 Bari, Italy; 2 Department of Civil, Environmental, Land, Building and Chemical Engineering, Dicatech, Polytechnic University of Bari, Via Edoardo Orabona 4, 70125 Bari, Italy

**Keywords:** lactic acid bacteria, exopolysaccharides, homopolysaccharides, dextran, sucrose, gene expression, nuclear magnetic resonance, monosaccharide composition

## Abstract

A metabolic feature of lactic acid bacteria (LAB) is the production of exopolysaccharides (EPSs), which have technological and functional properties of interest to the food sector. The present study focused on the characterization of the *Weissella cibaria* strain C43-11, a high EPS producer in the presence of sucrose, in comparison with a low-producing strain (C2-32), and on possible genetic regulatory elements responsible for the modulation of *dextransucrase* (*dsr*) genes expression. NMR analysis of the polymeric material produced by the C43-11 strain indicated the presence of dextran consisting mainly of a linear scaffold formed by α-(1–6) glycosidic linkages and a smaller amounts of branches derived from α-(1–2), α-(1–3), and α-(1–4) linkages. Molecular analysis of the *dsr* genes and the putative transcriptional promoters of the two strains showed differences in their regulatory regions. Such variations may have a role in the modulation of *dsr* expression levels in the presence of sucrose. The strong upregulation of the *dsr* gene in the C43-11 strain resulted in a high accumulation of EPS. This is the first report showing differences in the regulatory elements of the *dsr* gene in *W. cibaria* and indicates a new perspective of investigation to identify the regulatory mechanism of EPS production.

## 1. Introduction

Lactic acid bacteria (LAB) represent, together with yeasts, the microorganisms with the greatest number of applications in food preparation. The study of lactic populations has allowed the selection of strains with specific biotechnological characteristics that are useful in improving food quality. The metabolic activity of LAB activates a series of biochemical processes that decisively influence the rheological, nutritional, functional, sensory, and shelf life properties of the products [1,2,3]. LAB are frequently used in the fermentation of leavened doughs for the production of baked goods to improve the technological properties of these products, allowing the reduction/elimination of additives and improvers (“label free”) [4,5], and are also used to produce functional products (reduced salt/fat content, enhanced bioactive compounds, “yeast-free”, and increased protein content) [6,7,8,9,10].

One metabolic feature of LAB is the production of exopolysaccharides (EPSs) during their fermentation activity. These compounds can be homo- (HoPS) or hetero-polysaccharides (HePS) depending on the type of monosaccharides constituting the polymer chain, and can have several technological and functional properties. Generally, EPSs improve the technological and sensorial properties of food products, and can act as prebiotics since they are not digested by human enzymes and are available as a carbon source for probiotic bacteria in the intestine and/or can protect the probiotic bacteria during the gastrointestinal transit by acting as a barrier [11]. Moreover, high molecular weight (MW) EPSs are postbiotics with antiviral and immunomodulatory activities [12,13].

EPS include dextran, a homopolysaccharide with a linear structure containing at least 50% of D-glucopyranosyl units attached by α-(1–6) linkages with varying percentages of α-(1–2), α-(1–3) or α-(1–4) branches [14,15], which can be used in foods since it has been generally recognized as safe (GRAS) by the Food and Drug Administration. In recent years, dextrans have assumed technological importance because they can improve the rheology and texture of many fermented food products and baked goods. For example, the European Commission authorizes the use of a *Leuconostoc mesenteroides* preparation containing dextran as a food ingredient in bakery products [16] to improve the softness, crumb texture, and loaf volume. Dextrans can also replace gelling agents or fatty substances currently used as thickeners in bread because they influence the viscoelastic properties of the dough and positively affect bread consistency and shelf-life, thus satisfying consumer demands for reduced use of food additives [9,17,18,19,20,21,22]. In addition to improving food texture, dextrans also provide the product with functional properties, acting as prebiotics and having beneficial effects on human health (antioxidant activity, cholesterol reduction, possible immunomodulatory and antitumor activity) [23,24,25].

The LAB *Weissella* and *Leuconostoc* are known to produce significant amounts of EPS, and the *Weissella* genus produces particularly high levels of dextran [13,26,27].

Several studies have investigated the molecular processes involved in polysaccharide synthesis by LAB [28,29,30,31], and some authors have explored the genome of *W. cibaria* strains to examine some metabolic traits, in particular EPS production [32,33]. Genomic analysis showed the presence of a single extracellular dextransucrase (*dsr*) enzyme encoded by the *dsr* gene involved in the dextran synthesis. Dextransucrases are members of the GH70 family according to the CAZy classification (http://www.cazy.org (accessed on 22 February 2022); they synthesize dextran by hydrolysis of the glycosidic bond of sucrose, subsequently releasing energy to catalyze the transfer of D-glucopyranosyl residues to the growing chain of the polymer, and at the same time releasing fructose [34]. Several studies have characterized the Drs enzymes [14], but few investigate regulation of the expression of the *dsr* gene [27,35]. Yu et al. [35] examined the effect of sucrose on *W. cibaria* compared with *Lactobacillus* spp., while Hu and Gänzle [36] studied the effect of temperature on the expression of the *dsr* gene in *W. cibaria*.

Recently, *W. cibaria* strains isolated from wheat semolina have been characterized for their ability to produce EPS [37,38]. In particular, the C43-11 strain has been selected as the greatest EPS producer of those tested, and has been used to obtain liquid sourdoughs enriched in EPS suitable for use as a fat replacer in baked goods. These sourdoughs were used to make focaccia, allowing a 20% reduction in the amount of added fat and improving the technological characteristics of the final product [9].

Therefore, the present work (1) aimed to chemically characterize the EPS produced by C43-11 in comparison with the low-producing C2-32 strain; (2) to investigate the possible genetic factors responsible for EPS production by the two strains, and (3) to analyze the effect of sucrose on the expression of the *dsr* gene in the two strains.

## 2. Materials and Methods

### 2.1. Microorganisms and Growth Conditions

*W. cibaria* strains C43-11 and C2-32, isolated from wheat semolina [37], were cultured in MRS broth (Biolife Italiana S.r.l., Milan, Italy) and incubated at 30 °C. For long-term storage, stock cultures were prepared by mixing 8 mL of culture with 2 mL of Bacto glycerol (Difco, Becton Dickinson, Co., Sparks, MD, USA) and freezing 1 mL portions of this mixture at −80 °C. Cultures were subcultured twice and incubated at 30 °C for 24 h before use.

### 2.2. EPS Production in mMRS

To investigate the EPS production, the growth conditions used in Valerio et al. [38] were applied. Briefly, the modified MRS broth (mMRS) was obtained as reported in Minervini et al. [39] by adding fresh yeast extract (5% *w*/*v*) and maltose (1% *w*/*v*) to the commercial MRS medium (Biolife Italiana S.r.l., Milan, Italy), final pH 5.6. The commercial medium contained 20.0 g/L glucose, 5.0 g/L yeast extract, 2.0 g/L K_2_HPO_4_, 5.0 g sodium acetate, 0.2 g/L MgSO_4_·7H_2_O, 0.05 g/L MnSO_4_·H_2_O, 2.0 g/L di-ammonium citrate, 10.0 g/L beef extract, and 10.0 g/L peptone 1 g/L Tween^®^ 80. The medium, supplemented (mMRS + S) or not (mMRS) with 10% (*w*/*v*) sucrose, was inoculated at 4% (*v*/*v*) with 24 h cultures of each strain and incubated for 24 h at 30 °C. The cultures were labelled as follows: C43-11 + S and C2-32 + S when strains were grown in mMRS + S, and C43-11 and C2-32 when strains were grown in mMRS with no addition of sucrose. For each culture, three replicates were prepared. After 6 h, 10 h, and 24 h incubation, aliquots were taken to determine strain growth, EPS production, and RNA extraction. Cell enumeration was carried out by plate count on MRS agar after 48 h incubation at 30 °C.

### 2.3. Purification and Quantification of EPS

EPS purification from the bacterial cultures was performed as reported in Valerio et al. [38]. Briefly, liquid cultures of strains in mMRS + S and mMRS were heat treated at 100 °C for 15 min to inactivate enzymes responsible for EPS degradation [40] and centrifuged (9000× *g*, 10 min, 4 °C). The resulting supernatants were treated with three volumes of chilled ethanol 96–99% (*v*/*v*) and the solutions were stored overnight at 4 °C. The precipitated EPS were collected by centrifugation (11,325× *g*, 20 min, 4 °C), dissolved in distilled water, dialyzed (12–14 kDa) against distilled water at 4 °C for 48 h, and lyophilized. Lyophilized samples were used for EPS quantification, monosaccharide composition, and NMR analysis.

The concentration of EPS (g/L) was determined on the lyophilized samples rehydrated with distilled water at the initial volume according to the phenol-sulfuric method [41] using glucose as a standard (limit of detection, LOD: 0.078 g/L).

### 2.4. EPS Monosaccharide Composition

EPS monosaccharide composition was determined by acid hydrolysis of the lyophilized and rehydrated samples using 5% (*v*/*v*) perchloric acid (70%) at 100 °C for 5 h [42]. After filtration, hydrolyzed samples were analyzed using a HPLC Dionex DX500 system equipped with a GP50 gradient pump, an ED40 Electrochemical Detector in Pulsed Amperometric Detection (HPAEC-PAD), and Dionex PeakNet 5.11 chromatographic software. The chromatographic separation of sugars was carried out using a Dionex CarboPac PA1 column heated at 30 °C and a Carbopac PA1 guard column in isocratic mode with an elution of 150 mm NaOH at a flow rate of 1.0 mL/min [43]. Identification and quantification of the main monosaccharides was performed by integrating calibration peaks obtained from the relevant standards solutions of arabinose, glucose, fructose, and mannose, all purchased from Merck KGaA (Darmstadt, Germany).

### 2.5. NMR Spectroscopy

#### 2.5.1. Chemicals

3-(Trimethylsilyl)-2,2,3,3-tetradeutero-propionic acid sodium salt (TSP-*d_4_*, CAS No. 24493-21-8, 99%D, Armar Chemicals, Döttingen, Switzerland) and deuterium oxide (D_2_O, CAS. No. 7789-20-0, 99.86%D, Eurisotop, Saclay, France) were used for sample preparation. NMR tubes (Norell 509-UP 7) were provided by Norell, Landisville NJ, United States. The following compounds were used as a reference: dextran (analytical standard Set Mp 1000–400,000, CAS. No. 9004-54-0, Fluka, Sigma-Aldrich, Switzerland), fructooligosaccharides from chicory (FOS, Sigma Aldrich, St. Louis, MO, USA), inulin from chicory (CAS. No. 9005-80-5, Sigma Aldrich, St. Louis, MO, USA), L-(+)-arabinose (CAS No. 5328-37-0, ≥98%), D-(+)-galactose (CAS No. 59-23-4, ≥99%), D-(+)-glucose (CAS No. 50-99-7, ≥99.5%), D-(−)-fructose (CAS No. 57-48-7, ≥99%), D-(+)-mannose (CAS No. 3458-28-4, ≥99%), sucrose (CAS No. 57-50-1, ≥99.5%), and D-(+)-maltose monohydrate (CAS No. 6363-53-7, ≥99%).

#### 2.5.2. Sample Preparation

Twenty milligrams of dialyzed and lyophilized samples (C43-11 + S and C2-32 + S) prepared as described in paragraph 2.3, were added to 600 µL of TSP-*d_4_*/D_2_O solution [3-(trimethylsilyl) propionic-2,2,3,3-*d_4_* acid sodium salt in D_2_O (0.20%_w_)]. The resulting solution was vortexed (Advanced Vortex Mixer ZX3, VELP Scientifica Srl, Italy) for 5 min at 2500 rpm and then transferred to the NMR tube.

#### 2.5.3. NMR Experiment

NMR spectra were recorded through a Bruker Avance 400 MHz spectrometer equipped with a 5 mm inverse probe. Pulse lengths were calibrated before each experiment, and the probe tuning and matching were adjusted for each sample. The temperature of the probe was set at 303 K.

The chemical shifts are reported in ppm and referenced to the TSP-*d_4_* signal. The following acquisition parameters were used to record 1D ^1^H NOESY NMR: pulse program = noesygppr1d; size of fid (TD) = 64 K; spectral width (SW) = 20 ppm; transmitter offset = 4.7 ppm; power level for pre-saturation (pl9) calculated at 25 Hz based on 90° hard pulse; dummy scans (ds) = 4; number of scans (ns) = 128; acquisition time = 4.09 s; mixing time (d8) = 0.010 s; recycle delay (d1) = 5 s. The following acquisition parameters were used to record ^1^H NMR: pulse program = zg; size of fid (TD) = 64 K; spectral width (SW) = 20 ppm; transmitter offset = 5.00 ppm; dummy scans (ds) = 0; number of scans (ns) = 16; acquisition time = 4.09 s; recycle delay (d1) = 10.0 s. The following acquisition parameters were used to record ^13^C NMR: pulse program = zgpg; size of fid (TD) = 131 K; spectral width (SW) = 240 ppm; transmitter offset = 110 ppm; dummy scans (ds) = 4; number of scans (ns) = 512; acquisition time = 2.71 s; recycle delay (d1) = 10 s. The following acquisition parameters were used to record TOCSY spectra: pulse program = mlevphpr; size of fid (TD) = 4 K × 256; spectral width (SW) = 20 ppm; transmitter offset = 4.70 ppm; power level for pre-saturation (pl9) calculated at 25 Hz based on 90° hard pulse; dummy scans (ds) = 16; number of scans (ns) = 32; spin lock (d9) = 75.00 ms.

NMR raw data (Free Induction Decays, FIDs) were processed using the software MestReNova 11.0 (Mestrelab Research SL, Santiago de Compostela, Spain). The phase and the baseline were optimized manually to make the resonances in the Fourier transformed data positive for all of the resonances except for the residual signal of water, which was subjected to a pre-saturation step. The zero-order (PH0) and first-order (PH0) phase parameters were adjusted opportunely after having set the pivot parameter to the biggest peak in the spectrum. The baseline correction was applied to flatten the baseline on the Fourier transformed data. Multipoint baseline correction was adopted upon selecting the points corresponding to baseline regions (also known as control points) which were then used by the software to build a baseline model using the interpolation algorithm.

### 2.6. Analysis of the Dsr Gene

The genomic DNA of the two strains was extracted from 24 h MRS broth cultures using a Wizard Genomic DNA Purification Kit (Promega Corporation, Madison, WI, USA) according to the supplier′s specifications. DNA quality and quantity were assessed using an ND 1000 spectrophotometer (NanoDrop Technologies, Inc., Wilmington, NC, USA) and by 1% (*w*/*v*) agarose gel electrophoresis in TAE buffer. The complete *dsr* gene (ca. 4700 bp), which included the promoter region, was amplified using the primers dsr-full_F (5′-AACACGAAAAGACGCTTGCG-3′) and dsr-full_R (5′-TGAGTAGGGCTGGGGTACTG-3′). The primer pair was designed with Oligo Perfect v1.0 software (Invitrogen, Life Technologies, Grand Island, NY, USA) based on the *dsr* gene from the *W. cibaria* MG1 genome available in GenBank (accession number JWHU00000000).

Each 20 µL reaction mixture contained 10 µL of 2X InvitrogenTM Platinum SupereFi II PCR master mix (Thermo Fisher Scientific, Waltham, MA, USA), 0.5 µM of each primer and 1 µL (50 ng) of genomic DNA. The reaction mixtures were first incubated for 30 s at 98 °C, and then cycled for 30 cycles according to the following temperature profiles: 15 s at 98 °C, 15 s at an annealing temperature of 60 °C, 2 min at 72 °C, followed by a final extension for 5 min at 72 °C. The PCR products were analyzed by agarose gel electrophoresis (1.5%) in a TAE buffer stained with GelRed (Biotium, Hayward, CA, USA). The size of the amplified DNA fragments was estimated by comparison with a GelPilot 1 kb Plus Ladder (Qiagen GmbH, Hilden, Germany).

In addition to primers for full length amplification of the *dsr* gene, WcibDex fw (5′-GCATCTTTCAATACTTGAGG-3′) and WcibDex rev (5′-CATGACTTGTTGGCATAGC-3′) [44], dsr1a (5′-GGCATGCAAGTAATGGCTGA-3′), dsr1b (5′-ATTGATCCGGCACAAAATCAGCC-3′), dsr1c (5′-CTAGTCAAGACGTCGTGCTGGTC-3′), dsr1d (5′-TTCTGCCTGAACTTGTGGA-3′), and dsr1e (5′-ACAGTGCAAGTGCGGTAGTTGAT-3′) internal primers for gene sequencing were used.

Moreover, in order to amplify the promoter region, a primer pair was designed using Oligo Perfect v1.0 software (Invitrogen, Life Technologies, Grand Island, NY, USA) and based on the *dsr* sequence of *W. cibaria* C43.11. The promoter region (ca. 500 bp) was amplified using the primers dsrPROM_F (5′-CCGGCAGCTACCCAAAACTT-3′) and dsrPROM_R (5′-AYGGGGTGTCTGAATTRGGC-3′) and the PCR conditions described above. PCR products were purified with a QIA quick PCR Purification Kit (QIAGEN, Inc., Toronto, ON, Canada), and quantified by an ND 1000 spectrophotometer (NanoDrop Technologies, Inc., Wilmington, NC, USA). Sequencing of the *dsr* gene and promoter sequence was performed by using a Big Dye Terminator v3.1 Cycle Sequencing Kit (Applied Biosystems, Thermo Fisher Scientific, Waltham, MA, USA) on an ABI Prism 3100 Genetic Analyzer (Applied Biosystems Waltham, MA, USA).

Nucleotide sequences were aligned using Clustal Omega (https://www.ebi.ac.uk/Tools/msa/clustalo/ (accessed on 10 March 2022)) and compared to the *dsr* sequences of MG1 and SP7 (NCBI acc.n. NZ_JWHU01000000.1 and NZ_SDGK00000000.1) strains. Nucleotide sequences of the *dsr* gene and promoter region of C43-11 and C2-32 strains are available in the NCBI nucleotide sequence database under accession numbers ON791804, ON791805, ON791806, and ON791807.

The putative regulatory elements and start site for transcription were predicted in silico within the promoter region of the *dsr* gene by using PBROM [45].

### 2.7. Transcription Analysis

#### 2.7.1. RNA Extraction and cDNA Synthesis

At selected time points (T0, T6, T10, T24), total RNA was isolated from 2 mL aliquots of liquid cultures in mMRS + S and mMRS. At T0, RNA was extracted from 24 h broth cultures used for inoculation. Cells were harvested by centrifugation (4000× *g* for 5 min at 18 °C) and snap-frozen in liquid nitrogen. RNA was extracted using the CTAB-based method [46] and modified as follows. Cell pellets were re-suspended in 800 µL CTAB buffer and transferred into 2 mL tubes containing a loop full of acid-washed 425–600 μm glass beads (Sigma-Aldrich, St. Louis, MO, USA) and two iron balls (0.5 cm diameter Ø). The mechanical disruption of cells was performed in a Retsch MM301 mixer mill (Retsch Gmbh, Haan, Germany) at 15/s frequency for 30 s and cell lysates were centrifuged at 16,060× *g* for 10 min. RNA was purified from the supernatant using chloroform-isoamyl alcohol (24:1), as described by Wang and Stegemann [46].

Possible contamination by genomic DNA was removed by DNase I (Thermo Fisher Scientific, Waltham, MA, USA) digestion, according to the manufacturer’s instructions. RNA concentration and quality were determined using a NanoDrop ND-1000 spectrophotometer and RNA integrity was checked by gel electrophoresis on 1.5% (*w/v*) agarose gel stained with GelRed. The RNA was reverse transcribed to cDNA using the SuperScript IV First-Strand Synthesis System (Invitrogen, Waltham, MA, USA) following the manufacturer’s instructions. The cDNA was stored at −20 °C.

#### 2.7.2. Real-Time (RT) qPCR

RT-PCR was performed using a 7500 Fast Real-Time PCR System (Applied Biosystems Waltham, MA, USA) using Power Up SYBR Green Master Mix (Applied Biosystem, Foster City, CA, USA) according to the manufacturer’s instructions. The primers used for the quantification of *dsr* gene expression were GLUC_Rq_F (5′-AATGTTGACACGAGCGACCT-3′) and GLUC_Rq_R (5′-CCGGAAGCACCACTAGTTGT-3′). qPCR primers were designed using Oligo Perfect v1.0 software based on the *dsr* gene sequence of *W. cibaria* C43-11; they amplified a PCR product of 194 bp. Relative gene expression profiles of *dsr* at time points T0, T6, T10, and T24 were determined by comparison to the expression of the *recA* gene used as a reference gene. The primers used for the quantification of gene expression of the *recA* were Wcib-recA-F (5′-GTAACCCAGAAACGACGCCT-3′) and Wcib-recA-R (5′-AGATTTCAACTTCCACTTCACGG-3′). These primers, reported by Koirala et al. [30], were modified after comparison with the *recA* gene sequences of *W. cibaria* (NCBI acc.n. JWHU01000016.1). The PCR product of 195 bp was amplified. qPCR was performed in triplicate in a volume of 10 μL containing 5 μL of Power Up SYBR Green Master Mix, 200 nm of each primer, and 1 μL of cDNA. The conditions for amplification were as follows: 50 °C for 2 min, 95 °C for 2 min followed by 40 cycles of 95 °C for 15 s, and 60 °C for 40 s. A dissociation step was performed after the RT-PCR run. Relative expression (RQ) of the *dsr* gene at all-time points was calculated using the 2^−ΔΔCT^ method [47]. RQ values were calculated by comparison with *dsr* gene expression levels in mMRS.

### 2.8. Statistical Analysis

Data are presented as mean values ± standard deviations. Statistical analysis of the data was performed using STATISTICA 12.0 software (StatSoft, Inc., Tulsa, OK, USA). Data concerning strain loads, EPS production, and monosaccharides concentration were compared by applying a one-way ANOVA (*p* < 0.05), substituting data below the detection limit (LOD) with half of the LOD [48]. Significant differences (*p* < 0.05) among groups were determined by using a post hoc Tukey test.

## 3. Results

### 3.1. EPS Quantification and Characterization

#### 3.1.1. EPS Production

The EPS production by *W. cibaria* C43-11 and C2-32 was evaluated in mMRS with or without sucrose at 10% (*w*/*v*). Both strains grew in the presence of the two substrates (Table 1), reaching ca. 9 log cfu/mL after 24 h (*p* > 0.05), although strain C43-11 showed a higher cell number (*p* < 0.05) after 6 h in the presence of sucrose. EPS production for C43-11 was mainly observed in the presence of sucrose, reaching ca. 12 g/L after 24 h, while low amounts close to the LOD were produced by C43-11 with no added sucrose, and by strain C2-32 in both media (Table 1).

#### 3.1.2. Monosaccharide Composition

The HPAEC-PAD results (Table 2) showed that the polymeric material produced by C43-11 in the presence of sucrose (C43-11 + S) was composed of approximately 94% glucose, 1% mannose, and 5% fructose. This finding is consistent with the production of dextran as the major polysaccharide product. Differently, the strain C2-32 (C2-32 + S) produced a low amount of material consisting of approximately 54% glucose, 43% mannose, and 3% fructose. When no sucrose was added to the medium, the strain C43-11 produced a material consisting of 32% glucose, 63% mannose, and 5% fructose, while the strain C2-32 produced a polysaccharide with a composition of 47% glucose, 46% mannose, and 7% fructose (C2-32). Finally, no arabinose was detected in any of the cases.

#### 3.1.3. EPS Characterization (NMR)

Based on the findings described in the previous paragraph, the NMR study focused on the characterization of the polymeric material produced by C43-11 in the presence of sucrose (C43-11 + S, Table 2). The sample’s macromolecular profile was confirmed by the poor sharpness of the signals in the 1D ^1^H NOESY NMR spectra [49]. The absence of free monosaccharides (arabinose, galactose, glucose, fructose, and mannose) and disaccharides (sucrose and maltose) was confirmed by comparison with spectra of reference compounds (see Figure S1 in Supplementary Materials for further details). Dextran, inulin, and fructooligosaccharide (FOS) were considered among the possible EPSs produced by LAB species. Dextrans with different molecular weights (MW: 25, 80, and 270 kDa) were taken into consideration to disclose possible variations in the chemical shifts of the signals due to the size of the polysaccharide, but no significant differences in spectral features were recognized.

**Figure 1:**
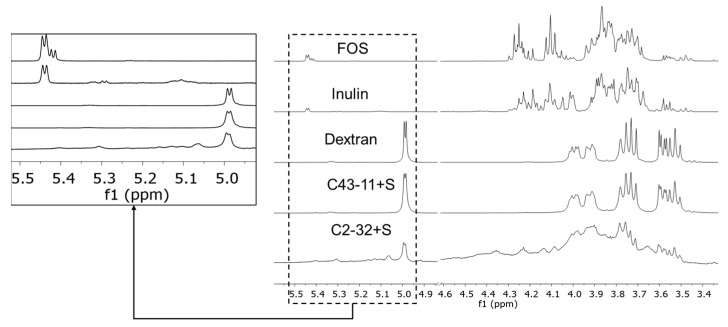
Typical 1D ^1^H NOESY NMR spectra (D_2_O, 400 MHz, 303 K) of fructooligosaccharide (FOS), inulin, dextran (MW 270 kDa) and purified samples from C43-11 + S and C2-32 + S.

In Figure 1, the 1D ^1^H NOESY NMR spectra of FOS, inulin, dextran (MW 270 kDa), and purified samples from C43-11 + S and C2-32 + S are reported.

**Figure 2:**
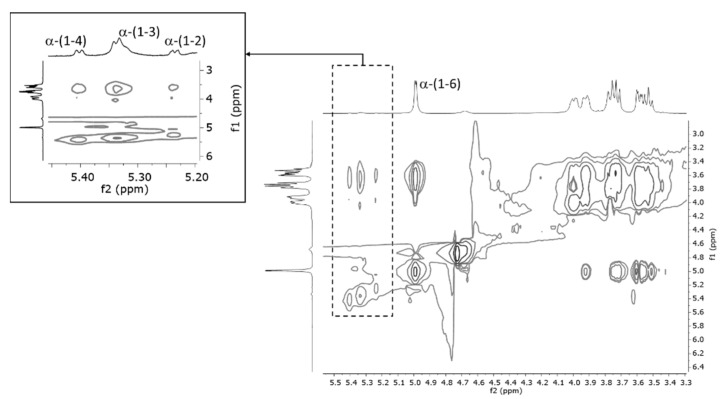
The TOCSY spectrum of EPS extracted from C43–11 + S recorded at 400 MHz in D_2_O at 303 K. Anomeric protons are labeled as α-(1–6), α-(1–2), α-(1–3), and α-(1–4).

Comparison of the spectra indicated that spectra labeled as dextran and C43-11 + S were almost superimposable. Thus, the signal pattern in the C43-11 + S spectrum could be mainly attributed to the glucose units constituting the polymeric chain of the dextran structure. Characteristic signals of anomeric hydrogen of dextran were identified in the region between 4.99 and 5.40 ppm, the most intense of which is attributable to α-(1–6) glycosidic linkage at 4.99 ppm. Weak signals at 5.40, 5.33, and 5.23 ppm were assigned to the anomeric hydrogen atoms involved in the α-(1–4), α-(1–3), and α-(1–2) linkages, respectively. These assignments were consistent with the correlations found in the ^1^H TOCSY experiment (Figure 2) which, in agreement with the data reported in the literature, supports the hypothesis that these hydrogens belong to the same polymeric scaffold [50]. Signal intensities of the anomeric hydrogens were consistent with a dextran structure predominantly consisting of α-(1–6) glycosidic linkage and a smaller amount of α-(1–4), α-(1–3) and α-(1–2) glycosidic linkages, in a ratio that was assessed at approximately 97 [α-(1–6)]: 3 [α-(1–4), α-(1–3), α-(1–2)].

The low fructose content revealed via analysis of the monosaccharide composition of sample C43–11 + S (5%, Table 2) was detected in the 1D ^1^H NOESY NMR spectrum in the form of very weak signals at 4.25 and 4.35 ppm (see Figure S2 in Supplementary Materials for further details). While no conclusive statements can be made based on their low intensities, the chemical shift values of these two signals may suggest the presence of heteropolymers similar to inulin or/and FOS.

**Figure 3:**
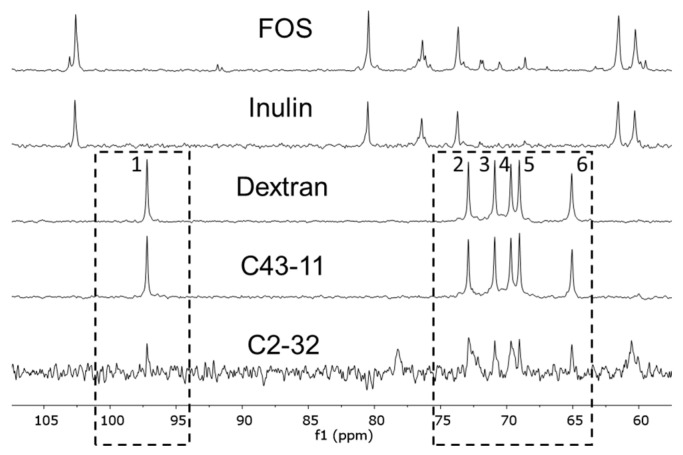
Comparison of typical ^13^C{^1^H} NMR spectra (D_2_O, 400 MHz, 303 K) of fructooligosaccharide (FOS), inulin, dextran (MW 270 kDa) and purified samples from C43-11 + S and C2-32 + S. The signals ascribed to the dextran structure are indicated in dashed squares.

The predominance of dextran in the EPS produced by C43-11 in the presence of sucrose was further confirmed by ^13^C{^1^H} NMR analysis. As represented in Figure 3, characteristic signals related to dextran were found at 97.2 (C-1), 72.89 (C-3), 70.89 (C-2), 69.70 (C-4), 69.07 (C-5), and 65.13 (C-6).

The ^1^H NMR signals in the spectrum of the material produced by C2-32 in the presence of sucrose (Figure 1) cannot be ascribed exclusively to dextran or to materials containing fructose (inulin and FOS). The presence of dextran can be supposed (Figure 3), but the low yield of the C2-32 + S sample did not allow a more detailed investigation of the structural features. Further investigations and appropriate growth conditions are needed to obtain information on the nature of the polysaccharides produced by the C2-32 strain.

### 3.2. Analysis of the Dsr Gene

Amplification of the *dsr* gene with primers *dsr*-full_F/*dsr*-full_R produced an amplicon of the expected length (4702 bp) that included the promoter region and the complete *dsr* gene of *W. cibaria* strains (4377 bp).

Pairwise sequence comparison of *dsr* genes of *W. cibaria* C43-11 and C2-32 strains revealed a 96% identity. The *dsr* genes of C43-11 and MG1, known as a high EPS producer, [33], showed 99% identity, while *dsr* genes of C2-32 and SP7 showed 98% identity (data not shown).

**Figure 4:**
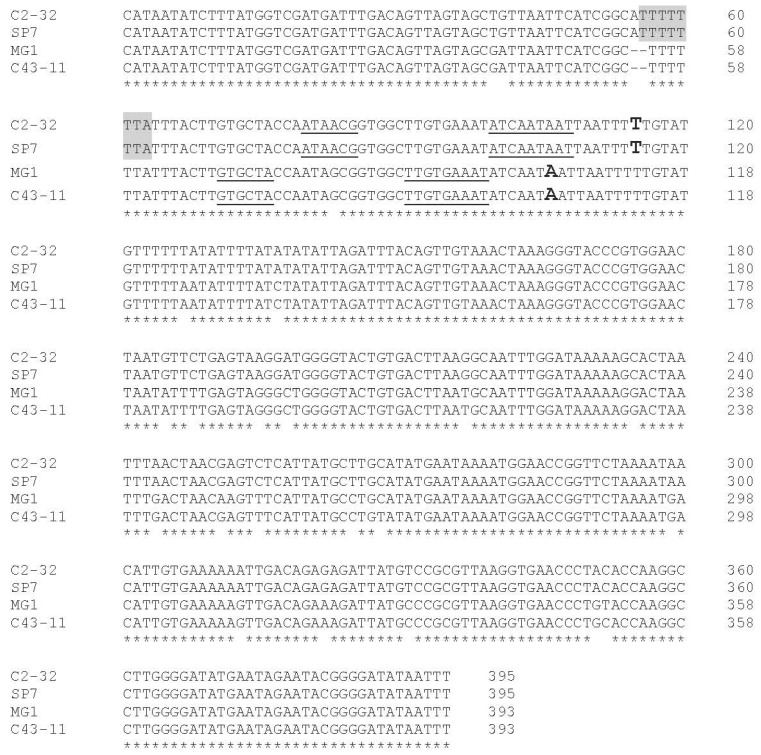
Alignment of the promoter regions of C2-32, C43-11, SP7, and MG1 strains with the in silico predicted −35 and −10 motifs (**underlined text**), transcriptional start sites (**bold letters**), and lexA (**light gray**) DNA-binding motif indicated.

The region upstream the *dsr* gene in both strains was determined by DNA sequencing and compared to the DNA sequence of those of *W. cibaria* MG1 and SP7 strains. The pairwise sequence comparison of the *dsr* gene promoter region of *W. cibaria* C43-11 and C2-32 strains showed a 94% identity (Figure 4).

DNA sequencing showed several nucleotide differences, in particular an AT insertion at position 55-56 of the C2-32 promoter sequence. A Blast N analysis against *W. cibaria* genomes available at the NCBI Genebank revealed the presence of this insertion also in other *W. cibaria* strains CSM1, CSM2, CMS3, CMU, and CXO-1 (data not shown). The sequence analysis for σ70-dependent DNA motif revealed a different pattern for −10 and −35 motifs. In detail, for strain C43-11 the transcriptional start site was predicted at an adenine (A) and the −10 (5′-TTGTGAAAT-3′) and −35 (5′-GTGCTA-3′) motifs were identified. Differently, for strain C2-32, the transcriptional start site was predicted at a thymine (T) and the −10 (5′-ATCAATAAT-3′) and −35 (5′-ATAACG-3′) motifs were identified.

**Figure 5:**
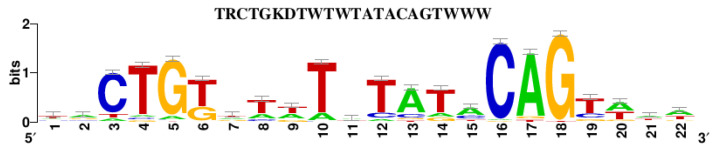
Position weight matrix (PWM) of LexA transcription factor binding (from RegulonDB—http://regulondb.ccg.unam.mx/ (accessed on 10 March 2022)).

Analysis of known transcription factor binding sites revealed the presence of a different pattern for C2-32 compared to C43-11. In particular, the C2-32 promoter region hosts additional lexA DNA-binding motif sites (Figure 4 and Figure 5) that are not present in the promoter region of the C43-11 *dsr* gene.

### 3.3. Transcription Analysis

**Figure 6:**
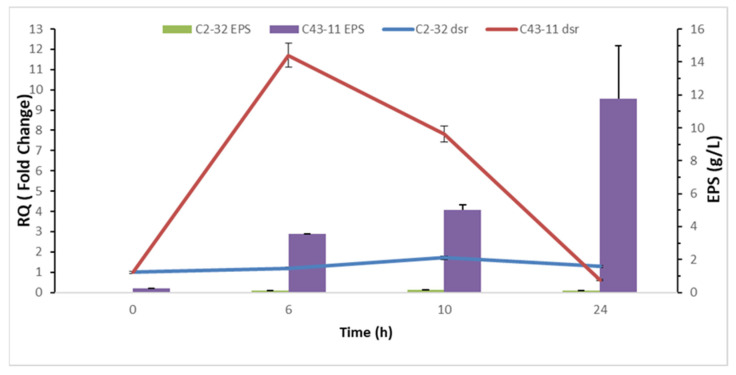
Relative Quantification (RQ, Fold Change = 2^−ΔΔCt^) of *dsr* gene expression in *W. cibaria* C43-11 and C2-32 grown in mMRS with sucrose (10% *w*/*w*). Reported RQ (lines) refer to *dsr* gene expression in mMRS with sucrose (mMRS + S) as compared to mMRS. RQ values are correlated with EPS production (g/L) for each strain in mMRS + S (bars).

To verify whether the differing EPS production between the two strains also resulted in a different expression level of dextransucrase, *dsr* gene expression was quantified at the mRNA abundance level. The relative expression of *dsr* was monitored in the presence and absence of sucrose during a 24 h incubation period and is shown in Figure 6. RT-qPCR demonstrated that *dsr* expression is strongly induced by the presence of sucrose only in C43-11, with the highest level occurring after 6 h (Fold Change > 10); subsequently, expression decreased up until 24 h. Conversely, for strain C2-32 the modulation of *dsr* expression did not respond to the supplementation of sucrose, showing only a slight upregulation after 10 h in the presence of sucrose (FC < 2, Figure 6).

## 4. Discussion

In recent years, microbial communities have been studied to select microorganisms with useful characteristics for the nutritional/functional improvement of fermented foods [31,51,52]. The use of LAB is considered an excellent tool for improving the nutritional/functional quality of foods via the production of bioactive compounds production, the bioavailability of dietary fibers and phytochemicals, and their technological and organoleptic characteristics, as well as the reduction of antinutritional factors [4,5]. Several studies have shown the positive impact of the EPS produced by LAB strains on the nutritional, technological, functional, and rheological features of foods [18,26,28,53]. In particular, *W. confusa* and *W. cibaria* have been studied for their ability to produce dextran [26].

In previous studies, the *W. cibaria* strain C43-11 was selected for its ability to produce high quantities of EPS [38], and was then used to produce a liquid sourdough applied in the production of focaccia bread as a fat replacer [9]. The current study has characterized the EPS produced by *W. cibaria* C43-11 and the *dsr* gene involved in dextran synthesis has been characterized. For this purpose, the *W. cibaria* C43-11 was compared with the strain C2-32, which belongs to the same species and has same origin but produces extremely low amounts of EPS [38]. Results showed that while both strains reached a cell load of about 9 log cfu/mL in mMRS containing sucrose or not, markedly higher EPS production was achieved only by C43-11 in the presence of sucrose. According to the results of Yu et al. [35], the difference in EPS production did not depend on the cell density but could be due to a higher gene expression.

The polymeric material was characterized by HPAEC-PAD and NMR analyses. HPAEC-PAD revealed a high glucose content in the hydrolysed samples of strain C43-11 grown in the presence of sucrose, supporting the hypothesis of dextran production. At the same time, the presence of fructose and mannose in addition to glucose after EPS hydrolysis was observed, suggesting the presence of oligosaccharides or heteropolysaccharides in low amounts. The presence of mannose could originate from the baker’s yeast used in the mMRS formulation, since it contains approximately 16% of this sugar [54], and could have been used as an acceptor in the synthesis of polymers other than dextran. Additionally, the release of cell wall components could be an effect of partial cell lysis caused by the heat treatment of the bacterial cultures during EPS purification. In particular, the monomers were detected in both *W. cibaria* culture filtrate samples, with or without sucrose and at similar concentrations, except for strain C43-11. In that case, glucose and fructose increased by approximately 100-fold and 30-fold, respectively, in the presence of sucrose. EPS production for strain C2-32 was scarce, and the amount and ratio of monomers was unchanged in the presence of sucrose, indicating that it did not influence the production of oligo- or heteropolysaccharides. Similarly, Yu et al. [35] studied the production of EPS by a *W. cibaria* strain isolated from kimchi in the presence of sucrose: NMR analysis showed the polymer to be α-1,6-dextran, and the ion chromatography revealed the presence of glucose, fructose and mannose at 62.1%, 34.2% and 3.8% in the MRS culture, and of 97% of glucose when the sucrose (2%, *w*/*w*) was added to the medium. Other authors also found low amounts of galactose, mannose, and glucose after acidic hydrolysis of the polymeric material obtained by a strain of dextran-producer *Leuconostoc mesenteroides* [27]. HePS can be produced by *Weissella* genus spp. and generally consist of mannose, glucose, fructose, arabinose, rhamnose, xylose, galactose, and in some cases containing *N*-acetyl-D-glucosamine and *N*-acetyl-D-galactosamine [26,55]. The production of polymers other than dextran by the *W. cibaria* strains could be further ascertained in a future study.

In agreement with the HPAEC-PAD results, NMR spectroscopy indicated that strain C43-11 produced dextran in the presence of sucrose. Dextran production was almost exclusive and was characterized by linear chains of glucose units bound predominantly by α-(1–6) glycosidic linkage. The signal assignment was in agreement with the data reported in the literature for dextran produced by wild and mutant strains of *Leuconostoc mesenteroides* [56]. It is known that the amount of α-(1–6) linkages in a dextran can vary from 50% to 97% of the total number of glycosidic linkages and the structural differences may depend on the strains and production conditions [50]. Moreover, Bounaix et al. [15,55] and Ahmed et al. [57] described the structure of dextran produced by *W. confusa* and *W. cibaria* strains containing 2.4–3.4% of α-(1–3) branch linkages. In the present study, traces of α-(1–4), α-(1–3) and α-(1–2) branched dextrans were also detected: approximately 1–3% compared to the α-(1–6) linkage. The formation of gluco-oligomers/polymers containing α-(1–4) and α-(1–2) has already been reported in the literature for LAB, like *Streptococcus*, *Lactobacillus*, and *Weissella* species incubated with sucrose and maltose [50,58].

To date, numerous studies have investigated the ability to produce EPS from the different bacterial isolates. The information gathered showed wide heterogeneity in EPS production capacity among strains. Based on the available information, the efficiency of the Dsr enzymes is influenced by different factors, including culture medium pH [42], the carbon source present in the growth medium [14], sucrose concentration [35], and temperature [36]. In particular, Bounaix et al. [15] and Besrour-Aouam et al. [14] indicated the optimal pH value of ca. 5.4 for Dsr activity. They also observed the influence of the carbon source (sucrose or glucose) on the synthesis of Dsr in different LAB species and *Weissella* strains and hypothesized that the regulation of *dsr* expression could depend on the species or strain. In fact, while *dsr* induction by sucrose is common in *Leuconostoc* spp. [15,59], Drs synthesis is reported as constitutive in other LAB, including *Lactobacillus reuteri* and *Streptococcus* spp. [60,61] and some *Weissella* strains [15]. In the present study, significant upregulation of the *dsr* gene was inducted in the presence of sucrose only in *W. cibaria* C43-11 strain, in agreement with the results obtained for *W. confusa* strain by Koirala et al. [30]. Moreover, Yu et al. [35] reported that EPS production was enhanced by increasing sucrose concentration in a *W. cibaria* strain; while Hu and Gänzle [36] and Besrour-Aouam et al. [27] reported the induction of the *dsr* gene by cold stress as well.

Although several studies have explored the factors affecting their differing ability to produce EPS, few have focused on the genetic characteristics of the regulatory elements of the *dsr* gene in *W. cibaria*. The present study focused on the expression of the *dsr* gene in two strains of *W. cibaria* to determine the regulatory elements that might be responsible for the difference in production capacity. In particular, the in silico analysis of the promoter region of the *dsr* gene in the two strains highlighted substantial differences concerning the predicted regulatory regions. Specifically, the AT insertion at position 55–56 of the C2-32 promoter sequence determined a shift in the transcriptional start site and −10 and −35 motifs. This evidence needs to be confirmed experimentally. The insertion generated a new DNA-binding motif, LexA, shared only with strain SP7 (100% match of the promoter region with C2-32 strains), and not with C43-11 or MG1. *W. cibaria* MG1 was the first and best studied EPS producer, also due to its promising potential for bakery and dairy applications [33,44,62,63], while the SP7 strain was reported as unable to produce EPS on modified MRS agar plates containing different carbohydrates [64]. LexA is known to repress the transcription of several genes involved in the cellular response to DNA damage or inhibition of DNA replication [65,66], as well as its synthesis [67]. This regulation is known as the SOS response [66]. To repress transcription, LexA blocks the access of RNA polymerase to target promoters [68]. These findings were consistent with expression pattern analysis of the *dsr* gene in the C2-32 strain. In the presence of sucrose, *dsr* gene expression was strongly upregulated in the C43-11 strain, while the expression of the gene was not modulated in C2-32. This shared behavior could be related to the presence in both strains of this additional DNA-binding motif not present in the C43-11 and MG1 strains. To the best of our knowledge, this is the first study reporting a difference in regulatory elements of *dsr* genes in *W. cibaria*. Our findings will require further investigation to verify whether dextran production in the C2-32 strain could be restored by stressor factors such as cold stress, anaerobic growth conditions, and oxidative stress.

## 5. Conclusions

This study investigated the EPS production ability of *W. cibaria* strain C43-11 in comparison with the low producing strain C2-32, highlighting the induction of EPS production in the presence of a high sucrose level only by the C43-11 strain. NMR analysis demonstrated the dextran production consisting of a linear chain of glucose units bound predominantly by α-(1–6) glycosidic linkage and in smaller amounts (1–3%) by α-(1–2), α-(1–3), and α-(1–4) glycosidic linkages. Molecular analysis revealed a differential regulation of the *dsr* gene involved in dextran biosynthesis. The in silico analysis of the *dsr* promoter region highlighted the presence of different stress-responsive regulatory elements in the low producer C2-32 strain that could be responsible for its low production rate in the tested conditions.

Therefore, this study provides new insights into the investigation of the molecular bases of differences in EPS production ability, and indicates a new perspective of investigation for the identification of the regulatory mechanism of EPS production. Furthermore, dextran produced by the C43-11 strain may be further investigated for its possible functional and technological properties in the food sector.

## Figures and Tables

**Table 1:** Cell count (log cfu/mL) and EPS production by *W. cibaria* C43-11 and C2-32 in mMRS with (+S) or without sucrose after 0 h, 6 h, 10 h, and 24 h incubation at 30 °C.

Culture	T0	T6	T10	T24
	**Cell Count (log cfu/mL)**			
C2-32	7.08 ±0.01 ^b^	7.20 ± 0.08 ^b^	7.57 ± 0.03 ^b^	8.52 ± 0.62 ^a^
C2-32 + S	7.07 ± 0.10 ^b^	7.40 ± 0.02 ^b^	7.50 ± 0.09 ^b^	8.59 ± 0.78 ^a^
C43-11	7.32 ± 0.03 ^a^	7.39 ± 0.01 ^b^	7.68 ± 0.03 ^b^	8.77 ± 0.78 ^a^
C43-11 + S	7.320 ± 0.06 ^a^	7.64 ± 0.06 ^a^	7.89 ± 0.02 ^a^	9.27 ± 0.29 ^a^
	**EPS (g/L)**			
C2-32	<LOD ^b,^*	<LOD ^c,^*	<LOD ^b,^*	<LOD ^b,^*
C2-32 + S	<LOD ^b,^*	0.12 ± 0.004 ^b^	0.14 ± 0.00 ^b^	0.13 ± 0.00 ^b^
C43-11	<LOD ^b,^*	<LOD ^c,^*	0.12 ± 0.00 ^b^	<LOD ^b,^*
C43-11 + S	0.23 ± 0.00 ^a^	3.55 ± 0.02 ^a^	5.02 ± 0.31 ^a^	11.74 ± 3.25 ^a^

Data are represented as mean ± standard deviation. ^a–c^ Values with different superscript letters within each column are significantly different (*p* < 0.05). cfu = colony forming unit. * LOD = 0.078 g/L.

**Table 2:** Glucose, mannose, and fructose (mg/L) produced by strains C43-11 and C2-32 in mMRS + S and mMRS bacterial cultures after quantified analysis by HPAEC-PAD.

Culture	Glucose	Mannose	Fructose
	**mg/L**		
**C43-11 + S**	16299.9 ± 2114.1 ^a^	203.4 ± 55.4 ^ab^	779.9 ± 2.7 ^a^
**C2-32 + S**	259.7 ± 75.9 ^b^	207.0 ± 19.0 ^ab^	13.8 ± 4.7 ^b^
**C43-11**	138.5 ± 23.3 ^b^	280.5 ± 23.3 ^a^	21.0 ± 8.5 ^b^
**C2-32**	125.8 ± 22.7 ^b^	124.9± 10.6 ^b^	20.0 ± 4.0 ^b^

Data are represented as mean ± standard deviation. ^a^^,b^ Values with different superscript letters within each column are significantly different (*p* < 0.05).

## Data Availability

The nucleotide sequences of the *dsr* gene and promoter region of C43-11 and C2-32 strains are available in the NCBI nucleotide sequence database under accession numbers ON791804; ON791805; ON791806; ON791807.

## Supplementary Materials

The following supporting information can be downloaded at: https://www.mdpi.com/article/10.3390/foods11182819/s1. Figure S1. Typical 1D 1H NOESY NMR spectra (D2O, 400 MHz, 303 K) of free mono- (arabinose, galactose, glucose, fructose, and mannose) and disaccharides (sucrose and maltose), and purified samples from C43-11 + S and C2-32 + S. Figure S2. Typical 1D 1H NOESY NMR spectra (D2O, 400 MHz, 303 K) of free mono- (arabinose, galactose, glucose, fructose, and mannose) and disaccharides (sucrose and maltose), and purified samples from C43-11 + S and C2-32 + S.
