# Supplementary material for: Characterization of Dextran Produced by the Food-Related Strain Weissella cibaria C43-11 and of the Relevant Dextransucrase Gene
Source: Foods. 2022 Sep 13;11(18):2819. doi: 10.3390/foods11182819 (PMC9498152; doi:10.3390/foods11182819)

Supplementary Materials

Characterization of dextran produced by the food-related strain *Weissella cibaria C43-11* and of the relevant *dextransucrase gene*

Palmira De Bellis [1,*,a], Massimo Ferrara [1,a], Anna Rita Bavaro [1], Vito Linsalata [1], Mariaelena Di Biase [1], Biagia Musio[2], Vito Gallo [2], Giuseppina Mulè [1] and Francesca Valerio [1]

[1] Institute of Sciences of Food Production (ISPA), National Research Council (CNR), Via G. Amendola 122/O, 70126 Bari, Italy
[2] Dipartimento di Ingegneria Civile, Ambientale, del Territorio, Edile e di Chimica (DICATECh), Politecnico di Bari, via Orabona 4, I-70125, Bari, Italy
* Correspondence: mirella.debellis@ispa.cnr.it; Tel.: +39-080-5929459; Fax: +39-080-5929374
[a] These authors contributed equally to this work.

Figure S1. Typical 1D ^{1}H NOESY NMR spectra (D_2O, 400 MHz, 303 K) of free mono- (arabinose, galactose, glucose, fructose, and mannose) and
disaccharides (sucrose and maltose), and purified samples from C43-11+S and C2-32+S.

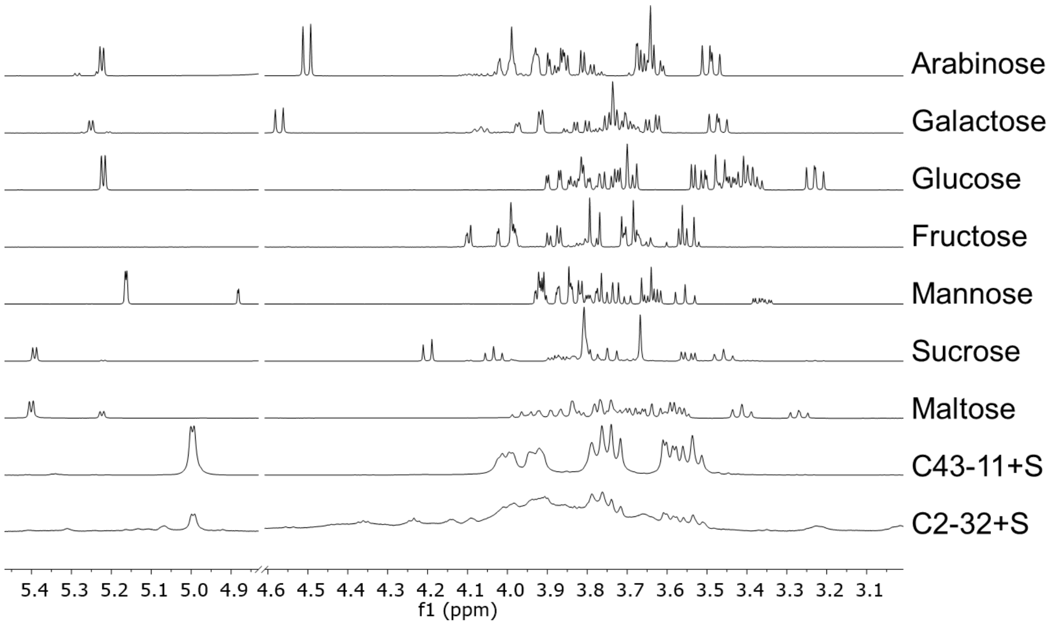

Figure S2. Typical 1D ^{1}H NOESY NMR spectra (D_2O, 400 MHz, 303 K) of free mono- (arabinose, galactose, glucose, fructose, and mannose) and disaccharides (sucrose and maltose), and purified samples from C43-11+S and C2-32+S.

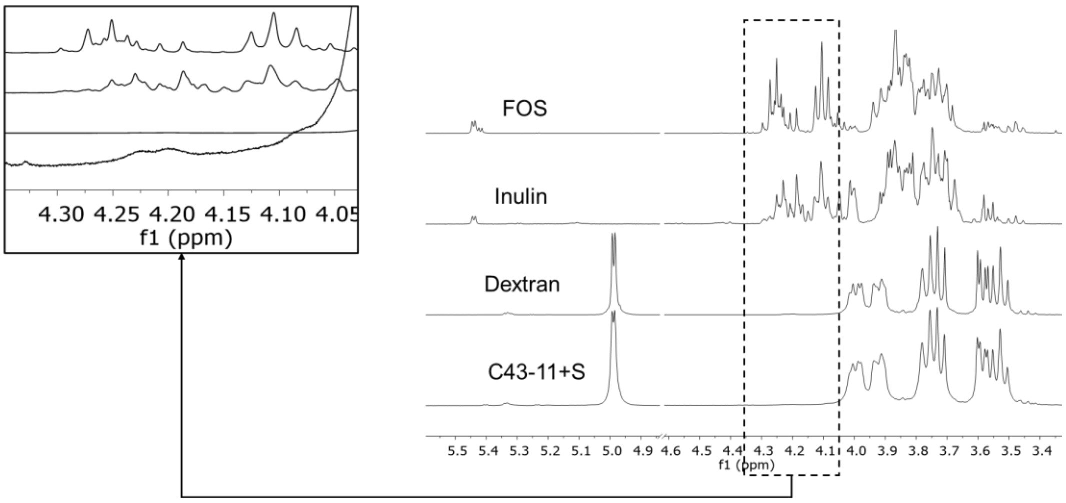

Supplement: Supplementary file 1 [file foods-11-02819-s001.zip › foods-1864089-supplementary.pdf]
